# Transcriptional Profiling and Transposon Mutagenesis Study of the Endophyte *Pantoea eucalypti* FBS135 Adapting to Nitrogen Starvation

**DOI:** 10.3390/ijms241814282

**Published:** 2023-09-19

**Authors:** Shengquan Huang, Xiuyu Zhang, Zongwen Song, Mati Ur Rahman, Ben Fan

**Affiliations:** 1 Department of Forestry, Nanjing Forestry University, Nanjing 210037, Chinamati@njfu.edu.cn (M.U.R.); 2 Department of Biology and Environment, Nanjing Forestry University, Nanjing 210037, China

**Keywords:** transcriptome, transposon library, endophyte, *Pantoea eucalypti*, nitrogen starvation, nitrogen limitation

## Abstract

The research on plant endophytes has been drawing a lot of attention in recent years. *Pantoea* belongs to a group of endophytes with plant growth-promoting activity and has been widely used in agricultural fields. In our earlier studies, *Pantoea eucalypti* FBS135 was isolated from healthy-growing *Pinus massoniana* and was able to promote pine growth. *P. eucalypti* FBS135 can grow under extremely low nitrogen conditions. To understand the mechanism of the low-nitrogen tolerance of this bacterium, the transcriptome of FBS135 in the absence of nitrogen was examined in this study. We found that FBS135 actively regulates its gene expression in response to nitrogen deficiency. Nearly half of the number (4475) of genes in FBS135 were differentially expressed under this condition, mostly downregulated, while it significantly upregulated many transportation-associated genes and some nitrogen metabolism-related genes. In the downregulated genes, the ribosome pathway-related ones were significantly enriched. Meanwhile, we constructed a Tn5 transposon library of FBS135, from which four genes involved in low-nitrogen tolerance were screened out, including the gene for the host-specific protein J, RNA polymerase σ factor RpoS, phosphoribosamine-glycine ligase, and serine acetyltransferase. Functional analysis of the genes revealed their potential roles in the adaptation to nitrogen limitation. The results obtained in this work shed light on the mechanism of endophytes represented by *P. eucalypti* FBS135, at the overall transcriptional level, to an environmentally limited nitrogen supply and provided a basis for further investigation on this topic.

## 1. Introduction

Nitrogen is one of the basic elements that make up bacterial cell macromolecules, such as proteins, nucleic acids, and cell walls. Nitrogen-mediated regulation in bacteria is a complex regulatory network involving many signal transduction and effector proteins. In response to nitrogen starvation, some bacteria directly activate the transcription of genes associated with scavenging and transport systems for alternative nitrogen sources, catabolism of nitrogenous compounds, and the glutamine biosynthesis pathway [1,2,3]. Moreover, the stringent response mediated by RelA (encoding the alarmone ppGpp synthetase) also plays a critical role in the bacterial adaptation to starvation and stress [4,5,6]. Under the condition of nitrogen-limitation stress, the nitrogen regulated (Ntr) response and the stringent reaction can be directly coupled [7,8]. These studies have been intensively performed in enterobacteria, whereas little is known about how an environmental *Pantoea* strain responds to nitrogen-limitation stress.

Endophyte refers to the bacteria, actinomyces, or fungi which live in normal plant organs or tissues and do not cause obvious symptoms of external infection in the host plants [9]. The endophyte is a natural component of the plant microecosystem. The co-evolution of endophytes and host plants has shaped the unique genetic characteristics and metabolites of endophytes. In the process of co-evolution, endophytes not only meet the needs of their own survival but also enhance the adaptability of the host to the external environment [10,11,12], thus playing an important role in the growth and development of the host plants and their resistance to adverse environments [13,14]. So far, endophytes such as *Bacillus*, *Pantoea, Pseudomonas*, and *Burkholderia* have been isolated from a variety of plants for extensive studies and potential development [15,16,17,18,19].

*Pantoea* is a genus of Gram-negative bacterial endophytes that are widely found in plant tissues, intercellular spaces, cortical cells, xylem, and soil [20,21,22]. *Pantoea* species can have beneficial effects on plants in a variety of ways [23,24]. For instance, firstly, *Pantoea* can secrete auxin (IAA), cytokinin (CTK), gibberellin (GA4), abscisic acid (ABA) [25] and induce plant system stress resistance [20,26] to directly regulate the plant’s growth. Secondly, *Pantoea* can produce a variety of antimicrobial substances to inhibit the growth of many pathogenic microorganisms, which thus indirectly promotes plant production [27,28,29,30,31,32]. Thirdly, *Pantoea* can help plant tissue cells to obtain necessary nutrient elements; for example, *Pantoea* spp. not only show the capability of dissolving insoluble phosphates [33,34] but also have the ability for biological nitrogen fixation [35,36,37]. In addition, *Pantoea* has been shown to induce systemic resistance and protection against phytopathogens in cultivated plants [38,39,40]. 

So far, research on *Pantoea* species has mainly focused on their effect on crops, with only a few reports on forestry applications [19]. In our previous studies, we isolated a pine growth-promoting bacterium, *P. eucalypti* FBS135, from the branches of *Pine massoniana*, which was identified as *Pantoea eucalypti* by phylogenetic analysis. We found that FBS135 had an obvious growth-promoting effect on pine growth [41] and a remarkable tolerance to low levels of nitrogen, as it can grow in different media containing no nitrogen sources. We have demonstrated that FBS135 does not possess the ability of nitrogen fixation [42]. We hypothesize that FBS135 may have specific genes/mechanisms involved in its tolerance to low nitrogen nutrition, therefore, in this study, we examined the transcriptome of FBS135 upon nitrogen deprivation and exploited a transposon library for the functional genes and metabolic pathways involved in nitrogen starvation. This work provided insight into the molecular basis of how a *Pantoea* endophyte adapts to nitrogen scarcity in the environment. 

## 2. Results and Analysis

### 2.1. FBS135 Transcriptomes of High Quality Were Obtained from Conditions with the Presence and Absence of NH_4_^+^

**Figure 1:**
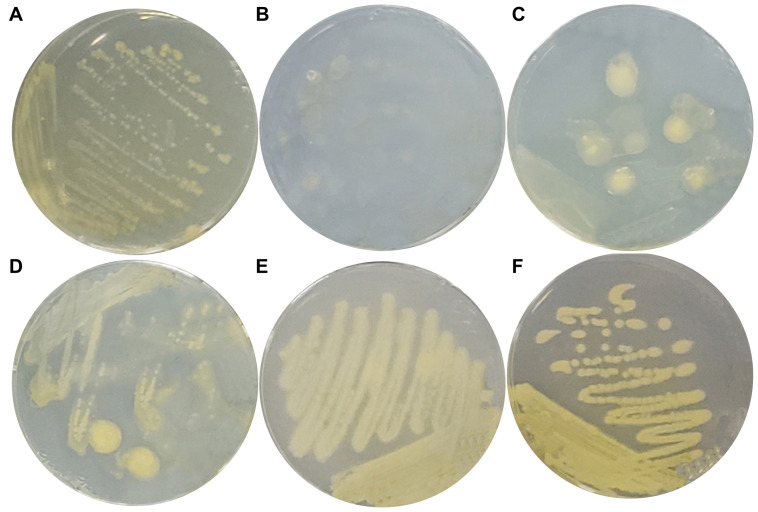
Growth of *P. eucalypti* FBS135 on solid media with different concentrations of nitrogen source. (**A**) LB (control with organic nitrogen); (**B**) nitrogen-free Ashby medium; (**C**) Ashby medium +0.2 g/L NH_4_Cl; (**D**) Ashby medium +1 g/L NH_4_Cl; (**E**) Ashby medium +8 g/L NH_4_Cl; and (**F**) Ashby medium +16g/L NH_4_Cl.

In previous studies, we have confirmed that *P. eucalypti* FBS135 can grow in/on nitrogen-free media [42]. To understand its gene expression in response to nitrogen limitation, we first designed a test to establish a suitable sample-collecting condition, which could be used in the transcriptome analysis experiment. We grew *P. eucalypti* FBS135 on nitrogen-free Ashby medium plates supplemented with inorganic nitrogen (NH_4_Cl) of different concentrations. FBS135 was able to grow on all the plates forming large colonies or lawns (Figure 1). With the gradual increase of the NH_4_Cl concentration, the color of the FBS135 colonies became yellowish, while their secretion of slime capsules decreased accordingly (Figure 1). When the concentration of NH_4_^+^ reached 8 g/L, the morphology of the FBS135 colonies looked similar to that on LB plates, with strong yellow pigment but a barely noticeable capsule. Under the condition of 1 g/L NH_4_Cl, FBS135 secreted a large amount of capsule, similar to the NH_4_Cl-free condition, which provided a common basis for the comparison of the FBS135 transcriptome under this condition with that under the condition of no additional NH_4_Cl; on the other hand, FBS135 secreted more yellow pigment, possibly carotene [41], than the NH_4_^+^-free condition, suggesting that this was a condition which could present a noticeable difference for the comparison. Therefore, we collected the cells of FBS135 grown on the nitrogen-free Ashby medium plates as well as the cells on the Ashby plates with 1 g/L NH_4_Cl for total RNA extraction and transcriptome sequencing.

The sequencing results showed that the proportion of clean reads in the six samples was above 96%, the number of N was below 15, and the low-quality reads were all 0%, indicating that the sequencing quality was good. The error rate of a single base site of each sample fluctuated at 0.2%, falling within the normal error range. The GC content of the same group was well correlated (Table 1). After raw data filtering, the clean bases of the transcriptome of each sample were all around 1 G. Their Q20 was greater than 98% and their Q30 was greater than 94%, higher than the normal level (Q20 > 95%, Q30 > 85%) (Table 1), indicating that the quality of the transcriptome sequencing was reliable.

After quality control, the high-quality sequences were mapped to the genome of *P. eucalypti* FBS135. The results showed that the total mapped reads were above 80%, while the multiple mapped reads ranged between 1% and 2%. The ratio of the number of reads mapped onto the genomic CDS region to the number of clean reads fell in the range of 65~82%, indicating a relatively high level of mapping.

### 2.2. P. eucalypti FBS135 Modulates Its Gene Transcription Adapting to Nitrogen Scarcity

**Figure 2:**
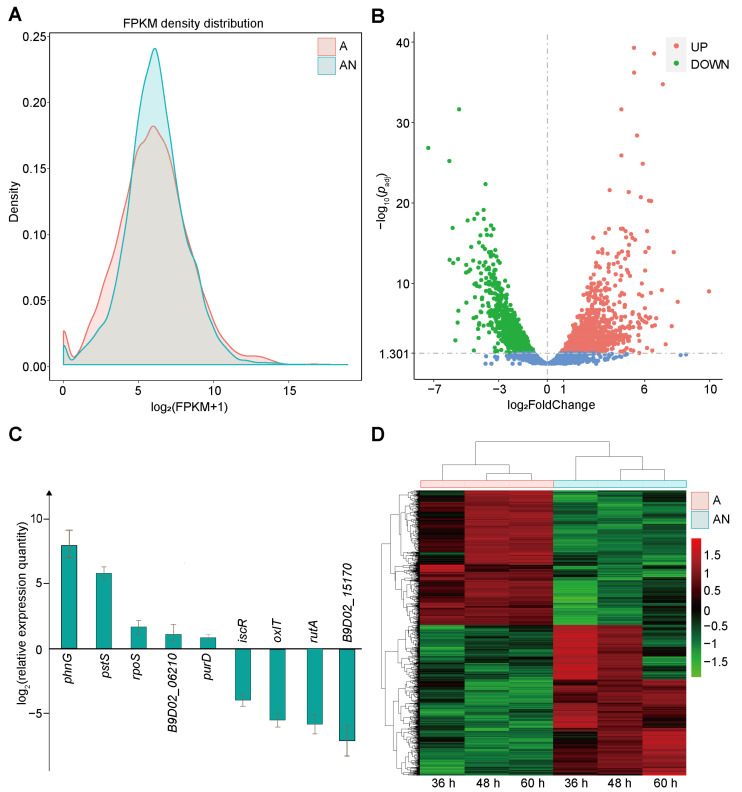
Effect of NH_4_^+^ addition on the general gene expression of *P. eucalypti* FBS135. (**A**) Distribution of gene expression levels of FBS135 without (**A**) and with (AN) NH_4_^+^ addition. (**B**) Volcano diagram of differentially expressed genes (DEGs) between the A condition and the AN condition. Upregulated genes in the A condition are indicated by red dots, while downregulated genes in the A condition are indicated by green dots. (**C**) qPCR verification of the DEGs of FBS135 detected via the transcriptome sequencing. (**D**) Hierarchical clustering of the DEGs under the A and the AN conditions.

The gene expression levels of *P. eucalypti* FBS135 were calculated based on the number of sequencing reads aligned to each transcript, and their distribution was normalized using FPKM. The results showed that the overall expression level of genes decreased when there was no NH_4_^+^ addition (Figure 2A), possibly indicating that many genes would not be expressed or under-expressed in an environment lacking nitrogen. 

Using *p*_adj_ < 0.05 and |FoldChange| > 2 as the screening criteria, we identified the differentially expressed genes (DEGs) using the negative binomial distribution model of DESeq. A total of 1936 genes were differentially expressed in *P. eucalypti* FBS135 (Figure 2B) (Supplementary Table S1). After adding NH_4_Cl, the expression of 1023 genes was upregulated, while the expression of 913 genes was downregulated (Figure 2B). Among the top five upregulated genes, three encode proteins for phosphonate metabolism with a 200–900 fold change, while among the top five downregulated genes, four encode for cold-shock proteins with a 60–160 fold change. We selected nine DEGs and verified their expression with qPCR, which gave a consistent fold change as the sequencing result (Figure 2C), indicating the transcriptome data were reliable. The genes were classified into two groups according to their expression levels in the different samples via hierarchical clustering analysis (Figure 2D). Three samples belonged to the group with the NH_4_Cl addition, while the other three belonged to the group with no NH_4_Cl addition. The expression of over 80% of genes varied significantly between groups but remained stable within one group, indicating a significant effect of nitrogen limitation on the gene transcription of FBS135. 

**Figure 3:**
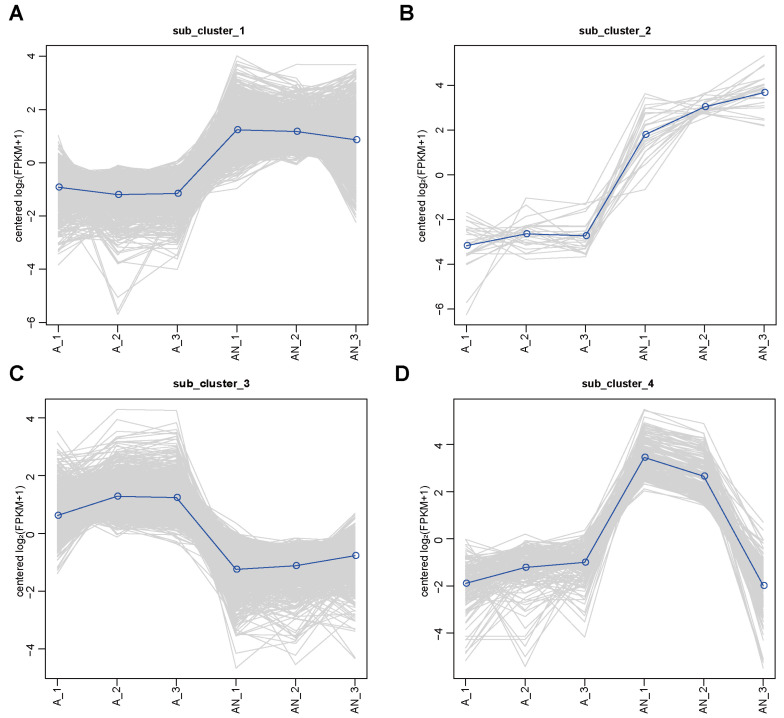
Different expression patterns of *P. eucalypti* FBS135 DEGs without (A) and with (AN) NH_4_^+^ addition. (**A**–**D**) Expression patterns of samples in different clusters. The samples from the A and the AN group harvested at 36, 48, and 60 h were numbered A_1, A_2, A_3, and AN_1, AN_2, AN_3, respectively.

Using the hierarchical clustering method, the 1936 DEGs were divided into four clusters, each of which had specific expression patterns among the six samples (Figure 3). Cluster 1 contained 832 genes, which were highly expressed in the presence of NH_4_^+^ (group AN) and lowly expressed in the absence of NH_4_^+^ (group A). The gene expression in either group showed a similar level at the three time points (36 h, 48 h, and 60 h) (Figure 3A). Notably, in this group ~18% of genes (64 out of 358) were enriched in the ABC transporter KEGG pathways, suggesting an enhanced transportation of substrates by *P. eucalypti* FBS135 responding to nitrogen starvation. Cluster 2 contained 25 genes, and most of the genes were also related to ABC transporters. They had low expression in the absence of NH_4_^+^ but high expression in the presence of NH_4_^+^. Unlike cluster 1, in the absence of NH_4_^+^ nitrogen, the expression level of the genes in this cluster showed an upward trend as culture time continued (Figure 3B). Cluster 3 contained 913 genes. In contrast to cluster 1, in this cluster the genes had low expression in the presence of nitrogen but high expression in the absence of nitrogen. However, similar to cluster 1, the expression under either condition showed a similar level across the three time points (Figure 3C). In cluster 3, ~23.5% of genes (65 out of 276) were enriched in the ribosome KEGG pathway. Ribosomes are the cellular factories responsible for producing proteins. Therefore, a large portion of downregulated genes were related to this pathway, which indicated a strongly reduced protein-making activity due to nitrogen limitation. Cluster 4 contained 166 genes, which were expressed at a similar level in the absence of NH_4_^+^ nitrogen. But in the presence of NH_4_^+^, their expression level increased by approximately 30 times at 36 h and 48 h, and then sharply dropped at 60 h to a similar level as under the condition of no NH_4_^+^ (Figure 3D). In general, it could be seen that when FBS135 was starved of a nitrogen source, the expression of half of its genes changed significantly, showing expression patterns over time.

### 2.3. Nitrogen Utilization-Related Genes of P. eucalypti FBS135 Were Significantly Downregulated in Expression upon Nitrogen Starvation

**Figure 4:**
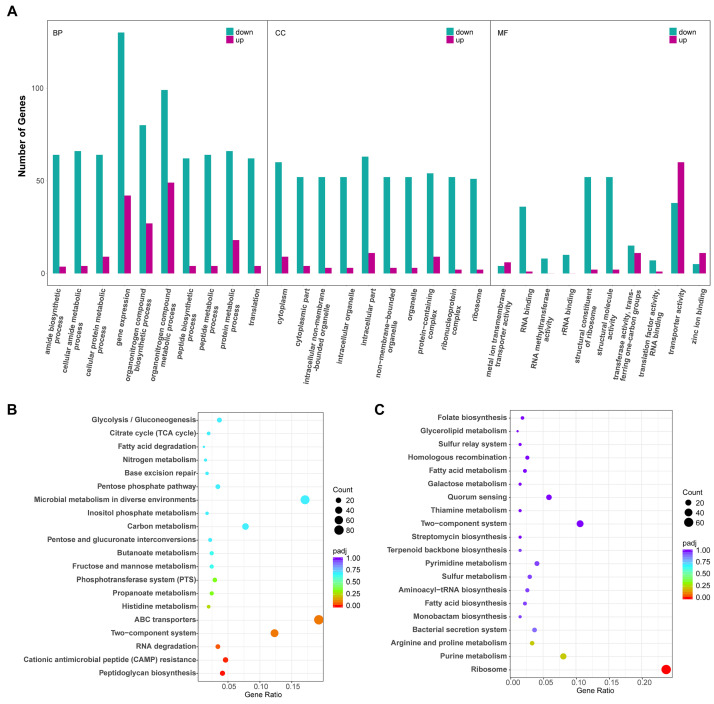
GO and KEGG enrichment analysis for the DEGs of *P. eucalypti* FBS135 under nitrogen deficiency. (**A**) 30 most enriched GO terms in the group of biological processes (BP), cellular component (CC), and molecular function (MF) under nitrogen deficiency. (**B**,**C**) 20 most enriched KEGG pathways of the upregulated (**B**) and downregulated (**C**) genes under nitrogen deficiency. Gene Ratio represents the ratio of the number of DEGs annotated to the KEGG pathway to the total number of DEGs.

Gene Ontology (GO) and the Kyoto Encyclopedia of Genes and Genomes (KEGG) pathway enrichment analyses were used to understand the functions of DEGs of *P. eucalypti* FBS135 adapting to nitrogen limitation. With *p*_adj_ < 0.05 as the threshold, there were 1841 DEGs enriched in different GO groups, including 780 (42.4%) categorized in Biological Process (BP), 733 (39.8%) categorized in Molecular Function (MF), and 328 (17.8%) categorized in Cellular Component (CC). When there was no NH_4_^+^ nitrogen, of the 30 most enriched GO terms, most were downregulated (Figure 4A), while only three GO terms in the BP group (gene expression, organonitrogen compound metabolic process, and organonitrogen compound biosynthetic process) and one term in the MF group (transport activity) contained more than 20 upregulated DEGs. Moreover, when there was no nitrogen, 80 genes were upregulated and 27 genes were downregulated in the process of organonitrogen compound biosynthesis; while 99 genes were upregulated and 49 genes were downregulated in the process of organonitrogen compound metabolism (Figure 4A). The result of the GO enrichment analysis showed that in the absence of a nitrogen source, the expression levels of FBS135 genes related to nitrogen source metabolism and nitrogen source utilization were changed significantly, mostly downregulated.

With *p*_adj_ < 0.05 as the threshold of significant enrichment, 696 DEGs were enriched in the KEGG pathways. The 20 most enriched KEGG pathways included the pathways for biosynthesis of secondary metabolites, ABC transporters, microbial metabolism in diverse environments, two-component system, and ribosome each containing more than 50 DEGs (Figure 4B,C). When deprived of nitrogen, the DEGs of FBS135 enriched in peptidoglycan biosynthesis and cationic antimicrobial peptide resistance were significantly downregulated (Figure 4B); whereas, the expression of ribosome-related genes was most significantly downregulated (Figure 4C), accounting for 24% of the total downregulated genes. The results were consistent with the findings of the GO enrichment analysis.

### 2.4. sRNAs Were Predicted with Potential Roles in the Adaptation to Nitrogen Starvation

The transcriptome sequencing reads can be assembled and compared with the annotated gene model to determine new unknown transcript regions, untranslated regions (UTRs), regulatory small non-coding RNAs (sRNAs), and single nucleotide polymorphisms (SNPs). Compared with the Nr library by Blastx (e-value = l × 10^−5^), a total of 458 new transcripts were predicted in the FBS135 genome (Supplementary Table S2). Based on the transcription information, 2936 5’-UTRs and 2915 3’-UTRs, and 120 sRNAs (50~492 bp) were predicted (Supplementary Table S3). In addition, the analysis revealed 18 sites with SNPs events and 74 sites with InDels events which were detected in the six samples (Supplementary Table S4). When NH_4_Cl was added, approximately half the number (59) of sRNAs were differentially expressed, with 36 of them upregulated and 23 downregulated (Supplementary Table S5). Among the differentially expressed sRNAs, sRNA00124 had the highest upregulation of 194-fold, and sRNA00261 had the highest downregulation of 65-fold. However, both of them remain unknown in function. So far non-coding regulatory RNAs have not been fully studied in most prokaryotes. These differentially expressed sRNAs in FBS135 may play an important role in the adaptation to nitrogen limitation and deserve further investigation in the future. 

### 2.5. Five Tn5 Insertion Mutants of P. eucalypti FBS135 Were Identified to Be Associated with Nitrogen Utilization

**Figure 5:**
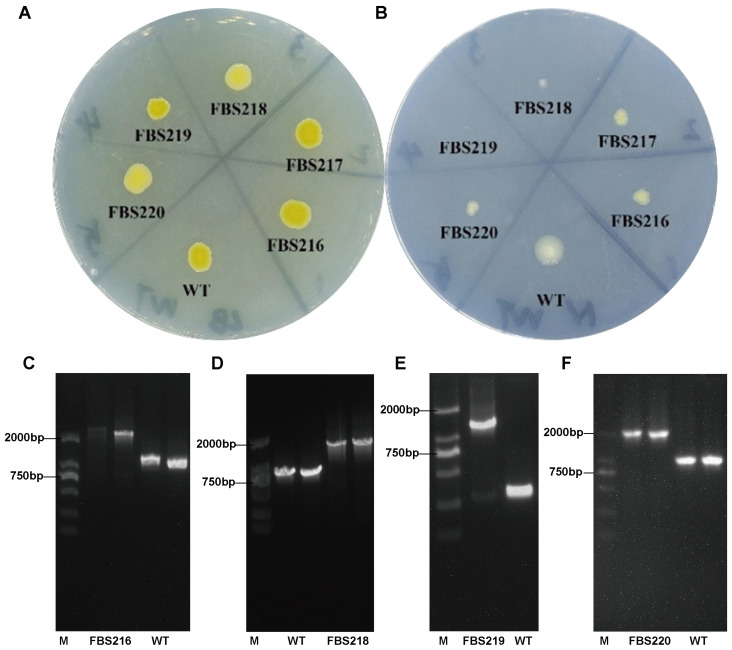
Five Tn5 insertion mutants of *P. eucalypti* FBS135 were defective in growth upon nitrogen scarcity. The growth status of the FBS135 wild-type and its Tn5 insertion mutants were grown on (**A**) LB medium plates and (**B**) nitrogen-free Ashby medium plates. (**C**–**F**) Confirmation via PCR of the products of the Tn5 insertion sites in four mutants. Lane M is the DNA marker DL2501 (Generay Biotech Co., Ltd., Shanghai, China).

In order to identify key genes related to the adaption of *P. eucalypti* FBS135 to nitrogen scarcity, we constructed a transposon mutant library using the method of three-parent hybrid transformation. After screening 5500 transport mutant strains, we discovered five strains that could not grow or grew poorly on nitrogen-free Ashby plates. We named them FBS216, FBS217, FBS218, FBS219, and FBS220. Similar to the FBS135 wild-type, the five mutant strains grew normally on LB agar plates forming yellow smooth colonies (Figure 5A). By contrast, FBS219 could not grow on nitrogen-free Ashby plates at all (Figure 5B). FBS216, FBS217, FBS218, and FBS220 could grow on the nitrogen-free plates after continuous streaking over 10 generations, however, their colonies were significantly reduced. Among the four of them, FBS218 grew the weakest, forming barely visible colonies (Figure 5B).

Using reverse PCR and random primer PCR technology, we successfully determined the Tn5 insertion sites in four mutants (FBS216, FBS218, FBS219, and FBS220) but failed to determine the Tn5 position in FBS217. In FBS219, Tn5 was inserted in the gene encoding serine acetyltransferase (SAT); in FBS216 and FBS220, Tn5 was inserted in the different positions of the gene for the host-specific protein J. Two Tn5 insertion sites were detected in FBS218, located in the RNA polymerase sigma factor RpoS gene and the phosphoribosylamine—glycine ligase gene.

In order to confirm the positioning result, we designed primers targeting the flanking sequences of Tn5 insertion sites in each mutant for PCR detection independently. The results of agarose gel electrophoresis showed that the DNA bands of the PCR products of each mutant differed from those of the FBS135 wild-type by ~1000 bp (Figure 5C–F), consistent with the length of the Tn5 transposon cassette (1066 bp). We further sequenced the PCR products, which showed complete consistency with the determined positions of Tn5.

### 2.6. Functional Analysis of the Tn5-Disrupted Genes Revealed Their Potential in the Adaption of P. eucalypti FBS135 to Nitrogen Scarcity

**Figure 6:**
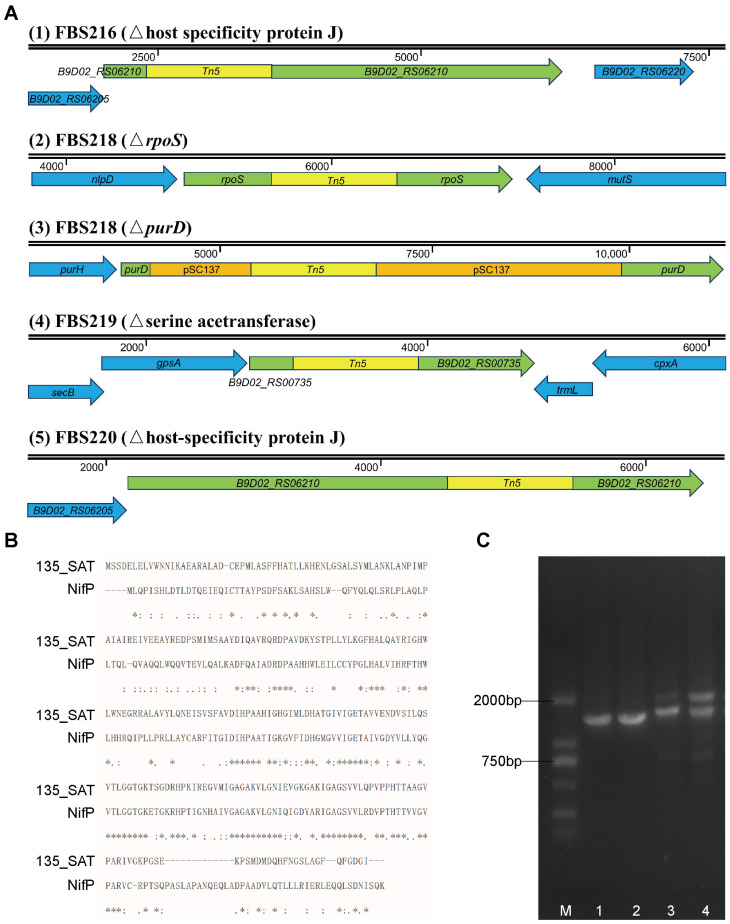
Localizations of Tn5 inserts in four transposon mutants of *P. eucalypti* FBS135 and the functional analysis of the mutant FBS218. (**A**) A schematic overview of Tn5 insertion sites in the four transposon mutants; (**B**) comparison of amino acid sequences between the serine acetyltransferase (SAT) of FBS218 and NifP encoded in nitrogen fixation gene cluster. “*” indicates a completely identical residue; “:” indicates a particularly similar residue; and “.” indicates a weakly similar residue. (**C**) Confirmation via PCR products of dual Tn5 insertion sites in FBS218. Lane M: the DNA marker DL2501 (Generay Biotech Co., Ltd., Shanghai, China); Lanes 1 and 2: PCR products using the genomic DNA of *P. eucalypti* FBS135 wild-type as a template; Lanes 3 and 4: PCR products using the genomic DNA of FBS218 as a template.

In order to understand the relationship of the disrupted genes by Tn5 to low nitrogen adaption, we analyzed their functions individually. The first attractive gene is the one interrupted by Tn5 in FBS219 (Figure 6A), which completely lost its ability to grow on a nitrogen-free solid medium. This gene is predicted to encode Ser acetyltransferase (SAT), which is responsible for the synthesis of O-acetylserine and critical for the biosynthesis of cysteine. Also, SAT is a key enzyme in the reaction process of sulfur assimilation. Through blast against the NCBI database, we found that this serine acetyltransferase of *P. eucalypti* FBS135 had 46.6% similarity to the NifP encoded in the nitrogen-fixing gene cluster of *Pseudanabaena* sp. ABRG5-3 (Figure 6B). NifP increases the synthesis rate of cysteine and methionine to optimize the expression activity of nitrogenase [43]. Although we have demonstrated that FBS135 cannot fix nitrogen, the presence of the SAT gene may contribute to its ability to adapt to extremely low nitrogen supply.

The gene encoding for the host-specific protein J was also intriguing since it was the one disrupted by Tn5 in both FBS216 and FBS220 (Figure 6A). Tn5 was inserted at the front part and the middle part of this gene in FBS216 and FBS220, respectively. The full length of the gene was 3210 bp, and its specific function is not yet fully determined. At present, it is only known that the protein is usually located at the end of a phage structure. Multiple tail assembly proteins and tail fiber assembly proteins, as well as cor-protein and peptidase P60, were found to be encoded upstream and downstream of the gene. The relationship between the host-specific protein J and low nitrogen tolerance has not been investigated. 

Two insertion sites of Tn5 were found in the mutant strain FBS218 (Figure 6A,C). The first insertion site was in the gene for RNA polymerase sigma factor RpoS, which regulates the expression of genes in response to environmental stresses [44]. The second site in FBS218 inserted by Tn5 was the gene *purD*, which encodes phosphoribosamine-glycine ligase. This gene has an important role in the biosynthesis of IMP and thus is involved in purine metabolic processes. Notably, the *purD* gene was not interrupted by the Tn5 transposon cassette, but the complete pSC137 plasmid sequence containing the Tn5 sequence (Figure 6A). This kind of insertion was verified via PCR and was not found in the other three mutants. Both *rpoS* and *purD* are important for the growth of FBS135, therefore, it remains unknown which gene is actually responsible for the failure of FBS218 to grow on nitrogen-free Ashby plates.

## 3. Discussion

In this study, we initially analyzed the transcriptome of *P. eucalypti* FBS135 in response to nitrogen scarcity. The RNA-Seq result showed that a total of 1936 genes changed significantly upon nitrogen starvation. The expression levels of genes related to nitrogen source metabolism and nitrogen source utilization were mostly downregulated, and the expression levels of genes related to ABC transporters, microbial metabolism in diverse environments, two-component systems, and ribosome pathways in different environments were most significantly changed. Expression changes of all the genes at the transcription level and their expression patterns represented a possible general fitness mechanism of these kinds of endophytes to nitrogen limitation. Furthermore, we identified four genes that may be related to the low-nitrogen adaption process through constructing and screening a transposon library, including the gene for the host-specific protein J, RNA polymerase σ factor RpoS, phosphoribosamine-glycine ligase, and serine acetyltransferase. These genes provide molecular targets for further investigations of the tolerance of the endophytes to nitrogen starvation. 

Endophytes are natural components of plant microecosystems. Due to their close relationship with plants, they have more direct and effective impact on plants than other associated microorganisms. However, the mechanism of how endophytic bacteria improve the resistance of host plants against various stresses is still poorly understood. Many endophytes, including some *Pantoea* species, have the ability to fix nitrogen [35,36,37]. The bacteria can convert molecular nitrogen into ammonia ions through the action of nitrogenase. The fixed nitrogen can be finally absorbed and utilized by the host plants, as shown in sugarcane, corn, rice, wheat, and other crops [35,36,37,45,46,47,48]. In recent years, some researchers also demonstrated that nitrogen-fixing endophytes in woody plants such as pine trees also act as and provide an additional nitrogen source, helping plants to form enormous amounts of biomass in barren soil [49,50,51,52]. 

In previous work, we isolated the endophyte *P. eucalypti* FBS135 from pine trees and demonstrated its growth-promoting effect on pine [41]. The ability of FBS135 to grow on/in a chemically defined medium with a lack of nitrogen source led us to speculate that FBS135 was able to fix nitrogen, which, however, was negated experimentally later [42]. However, a new question has thus been raised concerning how endophytes like FBS135 adapt to an extremely low supply of nitrogen in some environmental niches. Exploring the underlying mechanism may have great significance for understanding the relationship between endophytes and their host plants. 

Here we identified that a large number of *P. eucalypti* FBS135 genes altered their transcription under the condition of NH_4_^+^ nitrogen. Their expression patterns were linked to a stringent response to nitrogen starvation, which at least includes a general decreased expression of genes, typically those such as the ribosome-related genes. This change may reduce the nitrogen demand for some protein production and other unnecessary biological processes. Only a small number of genes were upregulated including those involved in transportation, which may promote the bacterial ability to acquire nitrogen from environments, and some genes for organonitrogen compound biosynthesis and metabolism. The regulation of the genes seems logical for the response of a bacterium to an environment lacking enough nitrogen supply. However, more principles and details could be scrutinized to understand the adaption mechanism of FBS135 to the stress of nitrogen limitation. 

The changed expression of many FBS135 genes may be associated with the gene *rpoS,* which we identified using the transposon library. According to the transcriptome data, *rpoS* was upregulated by approximately threefold upon nitrogen deficiency. As an RNA polymerase sigma factor, RpoS has the ability to control the expression of a large array of genes. RpoS has been reported to be involved in different environmental stresses such as acidic conditions, high osmotic pressure, oxidative conditions, and starvation conditions [53]. Importantly, it has been suggested that the association of the nitrogen control system and the gene expression system is regulated by *rpoS*, for example, in *Pseudomonas chlororaphis* the deletion of *rpoS* significantly reduces the utilization capacity of nitrogen sources such as alanine, proline, histidine, arginine, and urea [54].

Among the other three genes we determined using the Tn5 library, the one for SAT is most likely to be related to low-nitrogen adaption. Most nitrogen-fixing bacteria have six conserved genes, namely *nifH*, *nifD*, *nifK*, *nifE*, *nifN*, and *nifB*. These genes are used to encode nitrogenase and coenzyme factors, which are all necessary for nitrogen fixation [55]. None of these nitrogen-fixing genes were found in the genome of *P. eucalypti* FBS135. On the other hand, the SAT gene is phylogenetically similar to the *nifP* sequence, which is a part of the conserved nitrogen fixation gene cluster. *nifP* was originally identified in the ORF4 of *Azotobacter chroococcum* and showed 41% similarity to the *cysE* gene product of *Escherichia coli* [56]. *nifP*, *cysE*, and *nafG* are different gene forms of the key enzyme genes for SAT in diverse nitrogen-fixing bacteria. They are responsible for the biosynthesis of the iron–sulfur clusters of nitrogenase and play an important role in nitrogen fixation. It has been reported that nitrogenase activity decreased to 22% of the original level after the deletion of *nifP* in the non-heterocyclic cyanobacterium *Leptolyngbya boryana* [43]. Therefore, although SAT, like its homolog NifP, may not directly participate in biological nitrogen fixation, it may play a crucial role in nitrogen scarcity tolerance. 

Another gene that we identified encoding the host-specific protein J is unknown in function. Until now, in the NBCI database, there were only six whole genomes, including *P. eucalypti* FBS135, containing the genes encoding this protein. The other five strains were *P. vagans* PV989 [57], *P. ananatis* AJ13355 [58], *Pantoea* sp. At-9b, *Enterobacter* sp. SA187 [59], and *Metakosakonia* sp. MRY16-398 [60]. However, the disruption of the gene by Tn5 completely disabled the ability of FBS135 to grow on nitrogen-free Ashby agar plates. This also leaves an intriguing research topic for further investigation.

Endophytes including *Pantoea* species have been developed into various biocontrol agents [27,28,29,30,31,33]. Their close relationship with plants provides unique advantages in the application of them to promote the sustainable development of agriculture and forestry. Nevertheless, their biological characteristics need to be comprehensively elucidated before we can make the most of their potential. In terms of those species represented by *P. eucalypti* FBS135 and the work in this study, there are still many issues to be addressed in the future. For example, (i) the Tn5 insertion sites of FBS217 need to be determined; (ii) A suitable transformation method needs to be established for *P. eucalypti* FBS135 for further genetic manipulation; (iii) the *rpoS* and *purD* genes detected in FBS218 need to be separately knocked out and investigated individually; and (iv) complementation experiments need to be performed for the confirmation of the gene functions. It is expected that understanding the mechanism of their roles in low-nitrogen adaption may help to optimize parameters in the development and application of the bacteria as biological agents.

## 4. Materials and Methods

### 4.1. Strain Materials and Growth Conditions

*P. eucalypti* FBS135 was isolated from the shoots of *Pinus massoniana* as previously reported [41]. LB medium (Tryptone 10 g, Yeast extract 5 g, NaCl 10 g [61]) and Ashby medium (Sucrose 20 g, K_2_HPO_4_ 0.2 g, MgSO_4_·7H_2_O 0.2 g, NaCl 0.2 g, K_2_SO_4_ 0.1 g, CaCO_3_ 5 g, pH 7.4 ± 0.2) were routinely used. NH_4_Cl was added to the Ashby medium as indicated where necessary. 

Two experimental groups were set up for FBS135 cell collection and then their transcriptome comparison. In the first group (group A) the FBS135 wild-type was grown on nitrogen-free Ashby medium plates while in the second group (AN group) FBS135 was grown on Ashby medium plates with 1 g/L NH_4_Cl added. From the plates of both groups, cells were harvested at the time points 36 h, 48 h, and 60 h after inoculation by scraping the bacterial lawn off the plates. The samples collected from Group A at 36 h, 48 h, and 60 h were numbered A-1, A-2, and A-3, respectively, while the samples from Group AN at 36 h, 48 h, and 60 h were numbered AN-1, AN-2, and AN-3, respectively. For each sample, the collected cells were put into a 2 mL centrifuge tube and immediately frozen in liquid nitrogen, and stored at −80 °C before RNA extraction.

### 4.2. RNA Sequencing

After the total RNA was prepared, agarose gel electrophoresis (1.0%) was used to determine the integrity of RNA and DNA contamination. A Nano-Photometer spectrophotometer (Implen, Calabasas, CA, USA) and Agilent 2100 bioanalyzer (Agilent Technologies, San Francisco, CA, USA) were used to determine the purity and the integrity of RNAs, respectively. Subsequently, rRNAs were removed using Ribo-Zero Plus rRNA Depletion Kit (Lot No. 20037135) (Illumina, San Diego, CA, USA) to obtain the mRNA, which were then randomly fragmented into short fragments to construct strand-specific libraries. After the libraries passed the quality inspection, RNA sequencing was performed using the Illumina HiSeq sequencing platform at Novogene Technology Co., Ltd. (Beijing, China). 

### 4.3. Transcriptome Analysis

Expression levels of each transcript were calculated with the FPKM (fragments per kilobase of exon per million reads mapped) method using the HTseq version 0.6.1 [62]. The differential expressed genes were determined using the DESeq version 1.10.1 [63]. The ClusterProfile version 3.4.4 [64] was used for GO enrichment analysis and KEGG pathway enrichment analysis. For GO enrichment analysis, the enrichment factor was calculated using the ratio of the number of DEGs enriched in a GO term to the number of all genes in this GO term; for KEGG pathway enrichment analysis, the enrichment factor was calculated as the ratio of DEGs enriched in a KEGG pathway to the number of all genes in this KEGG pathway. 5′- or 3′-UTR and novel genes were predicted using the Rockhopper version 1.2.1 [65]. sRNAs were predicted using Rockhopper, the RNAfold version 2.0 [66], and the IntaRNA version 1.2.5 [67]. SNPs were predicted using the GATK version 3.5 [68].

### 4.4. Quantitative PCR

Total RNA was prepared using a previously described method [69]. Quantitative PCR was performed using the TB Green^®^ Premix Ex Taq kit (TaKaRa, Maebashi, Japan) and the StepOnePlus^TM^ Real-Time PCR System (ABI, Los Angeles, CA, USA) following the manufacturer’s instructions. The relative transcriptional level of each gene was quantified using the 2^−ΔΔCt^ method, with the housekeeping gene *gyrB* used as the internal standard. Sequences of all oligos used are provided in Supplementary Table S6.

### 4.5. Construction of the Mutant Library

The transposon mutant library of *P. eucalypti* FBS135 was constructed using the method of three-parent hybridization [70]. The suicide plasmid pSC137 containing the Tn5 cassette was transformed to FBS135 with the help of the pRK600. Those transformants which could grow on nutrient plates containing 5 μg/mL chloramphenicol and 5 μg/mL Erythromycin but could not grow on plates containing 100 μg/mL ampicillin were checked for their growing status. In total, 5500 mutant strains were screened for those which could not grow or grow weakly on nitrogen-free Ashby solid medium. The nitrogen-free NFDM solid medium (Sucrose 20 g, FeSO_4_·7H_2_O 0.015 g, MgSO_4_ 0.5 g, KH_2_PO_4_ 3.4 g, NaCl 0.01 g, Na_2_MoO_4_ 2H_2_O 0.005 g, K_2_HPO_4_ 12.06 g, pH to 7.0) was used for verification of the result obtained on Ashby plates.

### 4.6. Determination of the Gene Locations of Tn5 Transposon

The genomic DNA of mutant strains was extracted via the CTAB method [71]. The location of the Tn5 transposon was determined using inverse PCR [72] and random primer PCR [73] techniques. PCR products were sent to biocompanies for DNA sequencing. The local alignment tool Local Blast and the multiple sequence alignment tool (http://multalin.toulouse.inra.fr/multalin/multalin.html, accessed on 1 November 2018) were used to compare and analyze the sequencing results for Tn5 insertion.

### 4.7. Verification of the Locations of Tn5 Transposon

Oligos (Supplementary Table S6) were designed, targeting ~1000 bp upstream and downstream of the Tn5 insertion positions that have been determined above. The correctness of the Tn5 position was verified by the size of the PCR products compared to that of the *P. eucalypti* FBS135 wild-type and the DNA sequencing results of the PCR product.

## Figures and Tables

**Table 1:** Sample sequencing data quality summary of *P. eucalypti* FBS135.

Sample Name	Raw Reads	Clean Reads	Clean Bases	Error Rate	Q 20	Q 30	GC Content
AN_WT_1	7,797,428	7,673,596	1.16G	0.02	98.27	94.73	55.31
AN_WT_2	7,208,796	7,091,792	1.07G	0.02	98.37	94.99	53.84
AN_WT_3	7,801,876	7,697,252	1.16G	0.02	98.48	95.19	52.76
A_WT_1	7,637,382	7,550,282	1.14G	0.02	98.14	94.33	50.37
A_WT_2	7,858,328	7,767,674	1.17G	0.02	98.16	94.42	51.5
A_WT_3	7,435,342	7,338,626	1.11G	0.02	98.36	94.94	51.66

## Data Availability

Raw transcriptome data of the six samples were deposited in the SRA database (Sequence Read Archive, NCBI) with the accession numbers SRR24472222, SRR24472223, SRR24472224, SRR24472225, SRR24472226, and SRR24472227.

## Supplementary Materials

The supporting information can be downloaded at https://www.mdpi.com/article/10.3390/ijms241814282/s1.
